# 2-Amino-6-methyl-5-{5-[(naphthalen-2-yl­oxy)meth­yl]-1,3,4-oxadiazol-2-ylsulfan­yl}-4-(3-nitro­phen­yl)pyridine-3-carbonitrile

**DOI:** 10.1107/S1600536813005837

**Published:** 2013-03-06

**Authors:** Hoong-Kun Fun, C. S. Chidan Kumar, Prescilla Patrao, A. M. A. Khader, Balakrishna Kalluraya

**Affiliations:** a X-ray Crystallography Unit, School of Physics, Universiti Sains Malaysia, 11800 USM, Penang, Malaysia; b Department of Pharmaceutical Chemistry, College of Pharmacy, King Saud University, PO Box 2457, Riyadh 11451, Saudi Arabia; c Department of studies in Chemistry, Mangalore University, Mangalagangothri 574 199, India

## Abstract

The asymmetric unit of the title compound, C_26_H_18_N_6_O_4_S, contains two independent mol­ecules (*A* and *B*). The dihedral angles between the oxadiazole ring and naphthalene ring system are 42.59 (14) and 6.88 (14) Å in mol­ecules *A* and *B*, respectively. The dihedral angles between the pyridine and benzene rings in *A* and *B* are 65.53 (13 )and 87.67 (13) Å, respectively. In the crystal, mol­ecules *A* and *B* are linked through a pair of N—H⋯N hydrogen bonds involving one -NH_2_ group H atom and second pair of N—H⋯N hydrogen bonds involving the other -NH_2_ group H atom, forming an –*ABAB*– ribbon along [100] containing *R*_2_^2^(8) and *R*_2_^2^(12) ring motifs. These ribbons are further connected by weak C—H⋯N, C—H⋯O and C—H⋯π inter­actions, resulting in a three-dimensional network. The crystal studied was a non-merohedral twin with refined components 0.906 (1):0.094 (1).

## Related literature

For background to pyridine chemistry, see: Youngdale (1980 ▶, 1982 ▶); Todd (1970*a* ▶,*b* ▶); Lohaus *et al.* (1968 ▶, 1970 ▶); Gachet *et al.* (1995 ▶); Yao *et al.* (1994 ▶); Umemura *et al.* (1995 ▶). For background to 1,3,4-oxadiazole chemistry, see: Jin *et al.* (2006 ▶); Bhandari *et al.* (2008 ▶); Krasovskii *et al.* (2000 ▶); Mishra *et al.* (1995 ▶); Suman *et al.* (1979 ▶). For hydrogen-bond motifs, see: Bernstein *et al.* (1995 ▶). For standard bond-length data, see: Allen *et al.* (1987 ▶). For the stability of the temperature controller used for the data collection, see: Cosier & Glazer (1986 ▶).
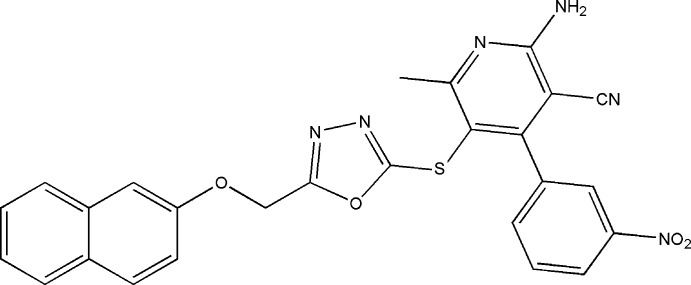


## Experimental

### 

#### Crystal data

C_26_H_18_N_6_O_4_S
*M* *_r_* = 510.52
Triclinic, 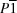

*a* = 9.6132 (9) Å
*b* = 9.8928 (10) Å
*c* = 25.724 (2) Å
α = 83.215 (2)°
β = 84.102 (2)°
γ = 72.564 (2)°
*V* = 2311.8 (4) Å^3^
*Z* = 4
Mo *K*α radiation
μ = 0.19 mm^−1^
*T* = 100 K
0.23 × 0.19 × 0.09 mm

#### Data collection

Bruker SMART APEXII CCD area-detector diffractometer
Absorption correction: multi-scan (*SADABS*; Bruker, 2009 ▶) *T*_min_ = 0.958, *T*_max_ = 0.983
9469 measured reflections
9469 independent reflections
8293 reflections with *I* > 2σ(*I*)
*R*_int_ = 0.000

#### Refinement

*R*[*F* ^2^ > 2σ(*F* ^2^)] = 0.056
*wR*(*F* ^2^) = 0.164
*S* = 1.20
9469 reflections
686 parameters
H atoms treated by a mixture of independent and constrained refinement
Δρ_max_ = 0.32 e Å^−3^
Δρ_min_ = −0.36 e Å^−3^

### 

Data collection: *APEX2* (Bruker, 2009 ▶); cell refinement: *SAINT* (Bruker, 2009 ▶); data reduction: *SAINT*; program(s) used to solve structure: *SHELXTL* (Sheldrick, 2008 ▶); program(s) used to refine structure: *SHELXTL*; molecular graphics: *SHELXTL*; software used to prepare material for publication: *SHELXTL* and *PLATON* (Spek, 2009 ▶).

## Supplementary Material

Crystal structure: contains datablock(s) global, I. DOI: 10.1107/S1600536813005837/lh5588sup1.cif

Structure factors: contains datablock(s) I. DOI: 10.1107/S1600536813005837/lh5588Isup2.hkl

Supplementary material file. DOI: 10.1107/S1600536813005837/lh5588Isup3.cml

Additional supplementary materials:  crystallographic information; 3D view; checkCIF report

## Figures and Tables

**Table 1:** Hydrogen-bond geometry (Å, °) *Cg*1, *Cg*2, *Cg*3 and *Cg*4 are the centroids of the C1*B*/C2*B*/C7*B*–C10*B*, C2*B*–C7*B*, C1*A*/C2*A*/C3*A*/C8*A*–C10*A* and O2*A*/C12*A*/N1*A*/N2*A*/C13*A* rings, respecively.

*D*—H⋯*A*	*D*—H	H⋯*A*	*D*⋯*A*	*D*—H⋯*A*
N4*A*—H2*NA*⋯N5*B* ^i^	0.86 (4)	2.21 (4)	3.025 (4)	158 (3)
N4*A*—H1*NA*⋯N3*B* ^ii^	0.84 (4)	2.23 (4)	3.056 (4)	165 (4)
N4*B*—H2*NB*⋯N3*A* ^ii^	0.92 (4)	2.08 (4)	2.991 (4)	174 (4)
N4*B*—H1*NB*⋯N5*A* ^i^	0.85 (5)	2.34 (5)	3.157 (4)	162 (4)
C11*A*—H11*B*⋯O1*B* ^iii^	0.99	2.36	3.332 (4)	168
C11*B*—H11*C*⋯O3*B* ^iv^	0.99	2.60	3.173 (4)	117
C21*B*—H21*B*⋯N2*A* ^v^	0.95	2.54	3.333 (4)	142
C22*A*—H22*A*⋯N1*B* ^vi^	0.95	2.60	3.540 (4)	169
C2*A*—H2*AA*⋯*Cg*1^vi^	0.95	2.78	3.492 (3)	133
C4*A*—H4*AA*⋯*Cg*2^vi^	0.95	2.62	3.457 (4)	147
C7*A*—H7*AA*⋯*Cg*1^iii^	0.95	2.80	3.557 (3)	138
C3*B*—H3*BA*⋯*Cg*3^vii^	0.95	2.67	3.440 (3)	138
C8*B*—H8*BA*⋯*Cg*3^viii^	0.95	2.83	3.599 (3)	139
C24*A*—H24*A*⋯*Cg*4	0.95	2.92	3.652 (3)	135

Symmetry codes: (i) 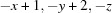; (ii) 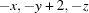; (iii) 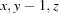; (iv) 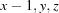; (v) 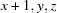; (vi) 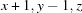; (vii) 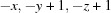; (viii) 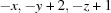.

**Fig. 1.**
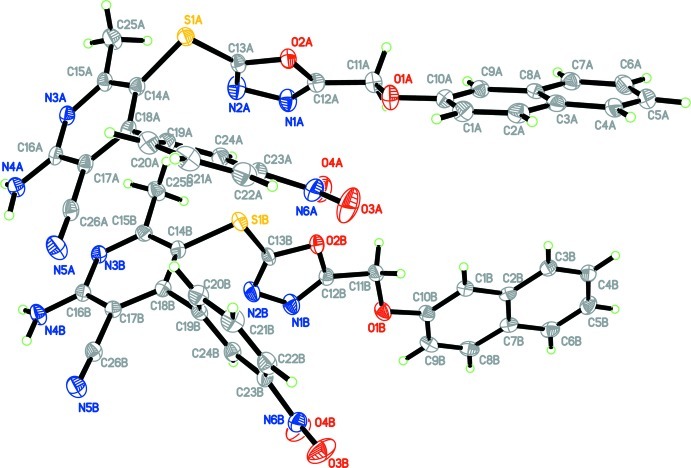
The asymmetric unit of the title compound with 50% probability displacement ellipsoids.

**Fig. 2.**
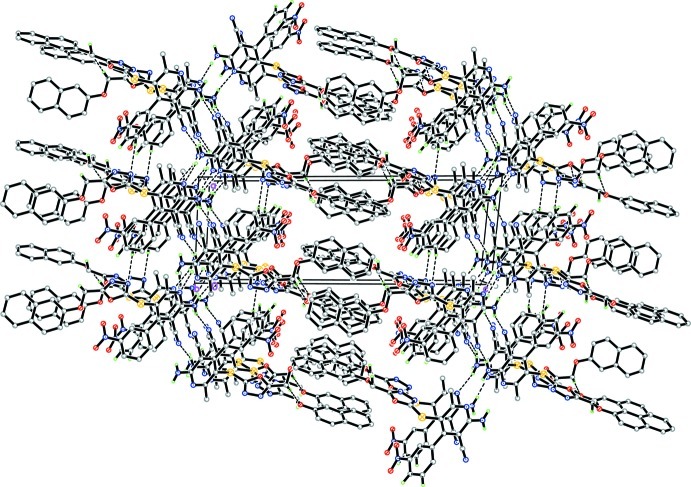
The crystal packing of the title compound. The H atoms not involved in the intermolecular interactions (dashed lines) have been omitted for clarity.

## supplementary crystallographic information

### Comment

Pyridine and its derivatives are of commercial interest and find application in medicinal drugs and in agricultural products such as herbicides,insecticides, fungicides, and plant growth regulators (picolines, lutidines, Timoprazole, and omeprazole). Pyridine derivatives have a wide range of biological activities being used as fungicidal, antibacterial, antifungal (Youngdale, 1980, 1982; Todd, 1970*a*,*b*), antimycotic (Lohaus *et al.*, 1968, 1970) and antidepressant agents (Gachet *et al.*, 1995), as well as thienopyridines being used as antithrombotic agents (Yao *et al.*, 1994; Umemura *et al.*, 1995) against platelet aggregation. Compounds containing a 1,3,4-oxadiazole ring have been reported to possess a broad spectrum of biological activities including insecticidal, antibacterial, anticancer, and anti-inflammatory (Jin *et al.*, 2006; Bhandari *et al.*, 2008; Krasovskii *et al.*, 2000) as well as antimycobacterial, analgesic, antipyretic and anticonvulsant properties (Mishra *et al.*, 1995; Suman *et al.*, 1979). The above observations prompted us to synthesize the title compound containing oxadiazole and amino pyridine carbonitrile groups and substituted pyridine scaffolds to determine its crystal structure.

The asymmetric unit of the title compound consists of two crystallographically independent, 5-(5-((naphthalen-6-yloxy)methyl)-1,3,4-oxadiazol-2-ylthio) -2-amino-6-methyl-4-(3-nitrophenyl)pyridine-3-carbonitrile molecules (A & B), as shown in Fig. 1. The bond lengths and angles of molecules A and B agree with each other and are within normal ranges for bond lengths (Allen *et al.*, 1987). The dihedral angles between pyridine rings (N3A/C14A–C18A)/ (N3B/C14B–C18B) and the benzene rings (C19A–C24A)/(C19B–C24B) are 65.53 (13) and 87.67 (13) Å, respectively. The central 1,3,4-oxadiazole ring system in both the molecules is essentially planar with maximum deviations of 0.007 (3) and 0.002 (3) %A respectively.

In the crystal structure (Fig. 2), molecule A is paired with molecule B via an N4—H···N3^ii^ hydrogen bonds (symmetry code in Table 1), involving the 4-amino group and the pyridine N1 atom and it is paired with another molecule of B through a pair N4—H···N5^i^ hydrogen bonds (symmetry code in Table 1), involving the 4-amino group and cyano N5 atom, forming *R*_2_^2^(8) and *R*_2_^2^(12) (Bernstein *et al.*, 1995) ring motifs. These hydrogen-bonded ABAB pairs lead to a extended ribbon structure. Theese ribbon are linked by weak C—H···N, C—H···O hydrogen bonds, resulting in a three-dimensional network. The crystal structure is further stabilized by C—H···π interactions (Table 1).

### Experimental

A mixture of malononitrile (10 mmol), substituted benzaldehyde (10 mmol), ammonium acetate (12.5 mmol) and 2-(5-((naphthalen-6-yloxy)methyl)-1,3,4-oxadiazol-2-ylthio)-1-(3-nitrophenyl)ethanone (8.33 mmol) in anhydrous benzene (25 ml) was refluxed for 18hrs, under nitrogen atm. Excess solvent was removed under reduced pressure. Residue was dissolved in ehtylacetate (50 ml) and washed successively with sodium bicarbonate solution (2 × 25 ml), water (2 × 25 ml), saturated brine solution (1 × 25 ml) and dried over anhydrous Na_2_SO_4_. Organic layer was evaporated to dryness and crude product was purified over silica using Dichloromethane/Methanol as eluent. Crystals suitable for X-ray studies were grown in a dimethylformamide solution (m.p:. 485 K).

### Refinement

N-bound H atoms were located in a difference Fourier map and were refined freely [refined N–H distance 0.86 (4), 0.84 (4), 0.92 (4) and 0.85 (4) Å]. The remaining hydrogen atoms were positioned geometrically [C–H = 0.95–0.99 Å] and were refined using a riding model, with *U*_iso_(H) = 1.2 *U*_eq_(C) or 1.5*U*_eq_(methyl C). A rotating-group model was used for the methyl group. The crystal used was a non-merohedral twin with refined components 0.906 (1):0.094 (1). The structure was refined using the HKLF 5 type input.

### Crystal data

**Table:**

C_26_H_18_N_6_O_4_S	*Z* = 4
*M**_r_* = 510.52	*F*(000) = 1056
Triclinic, *P*1	*D*_x_ = 1.467 Mg m^−^^3^
Hall symbol: -P 1	Melting point: 485 K
*a* = 9.6132 (9) Å	Mo *K*α radiation, λ = 0.71073 Å
*b* = 9.8928 (10) Å	Cell parameters from 9913 reflections
*c* = 25.724 (2) Å	θ = 2.2–31.8°
α = 83.215 (2)°	µ = 0.19 mm^−^^1^
β = 84.102 (2)°	*T* = 100 K
γ = 72.564 (2)°	Block, yellow
*V* = 2311.8 (4) Å^3^	0.23 × 0.19 × 0.09 mm

### Data collection

**Table:**

Bruker SMART APEXII CCD area-detector diffractometer	9469 independent reflections
Radiation source: fine-focus sealed tube	8293 reflections with *I* > 2σ(*I*)
Graphite monochromator	*R*_int_ = 0.000
φ and ω scans	θ_max_ = 26.5°, θ_min_ = 0.8°
Absorption correction: multi-scan (*SADABS*; Bruker, 2009)	*h* = −11→12
*T*_min_ = 0.958, *T*_max_ = 0.983	*k* = −12→12
9469 measured reflections	*l* = −8→32

### Refinement

**Table:**

Refinement on *F*^2^	Primary atom site location: structure-invariant direct methods
Least-squares matrix: full	Secondary atom site location: difference Fourier map
*R*[*F*^2^ > 2σ(*F*^2^)] = 0.056	Hydrogen site location: inferred from neighbouring sites
*wR*(*F*^2^) = 0.164	H atoms treated by a mixture of independent and constrained refinement
*S* = 1.20	*w* = 1/[σ^2^(*F*_o_^2^) + (0.0612*P*)^2^ + 3.203*P*] where *P* = (*F*_o_^2^ + 2*F*_c_^2^)/3
9469 reflections	(Δ/σ)_max_ < 0.001
686 parameters	Δρ_max_ = 0.32 e Å^−^^3^
0 restraints	Δρ_min_ = −0.36 e Å^−^^3^

### Special details

**Table:**

Experimental. The crystal was placed in the cold stream of an Oxford Cryosystems Cobra open-flow nitrogen cryostat (Cosier & Glazer, 1986) operating at 100.0 (1) K.
Geometry. All e.s.d.'s (except the e.s.d. in the dihedral angle between two l.s. planes) are estimated using the full covariance matrix. The cell e.s.d.'s are taken into account individually in the estimation of e.s.d.'s in distances, angles and torsion angles; correlations between e.s.d.'s in cell parameters are only used when they are defined by crystal symmetry. An approximate (isotropic) treatment of cell e.s.d.'s is used for estimating e.s.d.'s involving l.s. planes.
Refinement. Refinement of *F*^2^ against ALL reflections. The weighted *R*-factor *wR* and goodness of fit *S* are based on *F*^2^, conventional *R*-factors *R* are based on *F*, with *F* set to zero for negative *F*^2^. The threshold expression of *F*^2^ > σ(*F*^2^) is used only for calculating *R*-factors(gt) *etc*. and is not relevant to the choice of reflections for refinement. *R*-factors based on *F*^2^ are statistically about twice as large as those based on *F*, and *R*- factors based on ALL data will be even larger.

### Fractional atomic coordinates and isotropic or equivalent isotropic displacement parameters (Å^2^)

**Table:**

	*x*	*y*	*z*	*U*_iso_*/*U*_eq_	
S1A	0.16398 (8)	0.33559 (7)	0.14782 (3)	0.02095 (16)	
O1A	0.2069 (2)	0.3638 (3)	0.35113 (8)	0.0280 (5)	
O2A	0.1303 (2)	0.3595 (2)	0.25050 (8)	0.0213 (4)	
O3A	0.5886 (3)	0.4263 (4)	0.30149 (11)	0.0575 (8)	
O4A	0.3665 (3)	0.5428 (3)	0.28534 (9)	0.0396 (6)	
N1A	−0.0325 (3)	0.5693 (3)	0.25986 (10)	0.0277 (6)	
N2A	−0.0094 (3)	0.5480 (3)	0.20579 (10)	0.0287 (6)	
N3A	0.0759 (3)	0.6626 (3)	0.03544 (9)	0.0204 (5)	
N4A	0.2071 (3)	0.8008 (3)	−0.01105 (10)	0.0214 (5)	
N5A	0.5614 (3)	0.6973 (3)	0.03452 (11)	0.0288 (6)	
N6A	0.4891 (3)	0.4695 (3)	0.27254 (11)	0.0336 (6)	
C1A	0.3849 (3)	0.2575 (3)	0.41111 (12)	0.0252 (6)	
H1AA	0.4561	0.2476	0.3822	0.030*	
C2A	0.4263 (3)	0.2099 (3)	0.46061 (13)	0.0259 (6)	
H2AA	0.5267	0.1651	0.4658	0.031*	
C3A	0.3230 (3)	0.2255 (3)	0.50459 (12)	0.0236 (6)	
C4A	0.3647 (4)	0.1835 (3)	0.55661 (13)	0.0281 (7)	
H4AA	0.4649	0.1416	0.5629	0.034*	
C5A	0.2605 (4)	0.2033 (3)	0.59803 (13)	0.0316 (7)	
H5AA	0.2895	0.1772	0.6329	0.038*	
C6A	0.1123 (4)	0.2614 (3)	0.58938 (13)	0.0294 (7)	
H6AA	0.0412	0.2709	0.6183	0.035*	
C7A	0.0686 (3)	0.3049 (3)	0.53921 (12)	0.0253 (6)	
H7AA	−0.0324	0.3450	0.5338	0.030*	
C8A	0.1731 (3)	0.2905 (3)	0.49567 (12)	0.0209 (6)	
C9A	0.1316 (3)	0.3385 (3)	0.44375 (12)	0.0216 (6)	
H9AA	0.0316	0.3822	0.4375	0.026*	
C10A	0.2355 (3)	0.3217 (3)	0.40290 (12)	0.0232 (6)	
C11A	0.0578 (3)	0.4200 (3)	0.34090 (12)	0.0245 (6)	
H11A	0.0122	0.5059	0.3597	0.029*	
H11B	0.0050	0.3488	0.3531	0.029*	
C12A	0.0495 (3)	0.4566 (3)	0.28371 (11)	0.0217 (6)	
C13A	0.0851 (3)	0.4250 (3)	0.20303 (11)	0.0194 (6)	
C14A	0.1813 (3)	0.4818 (3)	0.10292 (11)	0.0198 (6)	
C15A	0.0658 (3)	0.5560 (3)	0.07162 (11)	0.0210 (6)	
C16A	0.2025 (3)	0.6962 (3)	0.02677 (10)	0.0188 (5)	
C17A	0.3244 (3)	0.6238 (3)	0.05665 (11)	0.0192 (6)	
C18A	0.3120 (3)	0.5182 (3)	0.09630 (10)	0.0182 (5)	
C19A	0.4320 (3)	0.4553 (3)	0.13243 (11)	0.0196 (6)	
C20A	0.5712 (3)	0.3765 (3)	0.11430 (13)	0.0259 (6)	
H20A	0.5901	0.3577	0.0785	0.031*	
C21A	0.6824 (3)	0.3254 (3)	0.14864 (13)	0.0290 (7)	
H21A	0.7764	0.2706	0.1361	0.035*	
C22A	0.6573 (3)	0.3536 (3)	0.20104 (14)	0.0300 (7)	
H22A	0.7330	0.3201	0.2245	0.036*	
C23A	0.5191 (3)	0.4318 (3)	0.21772 (12)	0.0252 (6)	
C24A	0.4061 (3)	0.4836 (3)	0.18467 (11)	0.0205 (6)	
H24A	0.3122	0.5376	0.1976	0.025*	
C25A	−0.0770 (3)	0.5216 (4)	0.07700 (13)	0.0288 (7)	
H25A	−0.1439	0.5870	0.0527	0.043*	
H25B	−0.1200	0.5317	0.1131	0.043*	
H25C	−0.0605	0.4235	0.0687	0.043*	
C26A	0.4569 (3)	0.6636 (3)	0.04550 (11)	0.0213 (6)	
S1B	0.12684 (8)	0.83421 (8)	0.21600 (3)	0.02183 (17)	
O1B	−0.1561 (2)	1.2195 (2)	0.39059 (8)	0.0265 (5)	
O2B	−0.0028 (2)	0.9464 (2)	0.30307 (8)	0.0208 (4)	
O3B	0.6655 (3)	1.0426 (3)	0.29656 (11)	0.0454 (7)	
O4B	0.4448 (3)	1.1689 (3)	0.28241 (10)	0.0377 (6)	
N1B	−0.0584 (3)	1.1801 (3)	0.28436 (10)	0.0276 (6)	
N2B	0.0113 (3)	1.1138 (3)	0.23840 (10)	0.0262 (6)	
N3B	0.0583 (2)	1.0526 (3)	0.07428 (9)	0.0192 (5)	
N4B	0.1950 (3)	1.1622 (3)	0.01522 (10)	0.0247 (5)	
N5B	0.5528 (3)	1.0738 (3)	0.05879 (10)	0.0270 (6)	
N6B	0.5503 (3)	1.0651 (3)	0.27607 (10)	0.0266 (6)	
C1B	−0.1881 (3)	1.1234 (3)	0.48129 (11)	0.0206 (6)	
H1BA	−0.1440	1.0282	0.4729	0.025*	
C2B	−0.2398 (3)	1.1516 (3)	0.53383 (12)	0.0212 (6)	
C3B	−0.2306 (3)	1.0404 (3)	0.57422 (12)	0.0230 (6)	
H3BA	−0.1890	0.9447	0.5662	0.028*	
C4B	−0.2807 (3)	1.0679 (3)	0.62501 (12)	0.0256 (6)	
H4BA	−0.2746	0.9916	0.6517	0.031*	
C5B	−0.3413 (4)	1.2102 (4)	0.63744 (12)	0.0291 (7)	
H5BA	−0.3744	1.2295	0.6726	0.035*	
C6B	−0.3525 (3)	1.3201 (3)	0.59898 (12)	0.0265 (6)	
H6BA	−0.3935	1.4152	0.6078	0.032*	
C7B	−0.3041 (3)	1.2946 (3)	0.54617 (12)	0.0225 (6)	
C8B	−0.3159 (3)	1.4046 (3)	0.50515 (13)	0.0262 (6)	
H8BA	−0.3596	1.5004	0.5129	0.031*	
C9B	−0.2666 (3)	1.3773 (3)	0.45474 (13)	0.0253 (6)	
H9BA	−0.2751	1.4533	0.4278	0.030*	
C10B	−0.2024 (3)	1.2343 (3)	0.44277 (12)	0.0223 (6)	
C11B	−0.1193 (3)	1.0798 (3)	0.37579 (11)	0.0231 (6)	
H11C	−0.2067	1.0450	0.3806	0.028*	
H11D	−0.0439	1.0157	0.3981	0.028*	
C12B	−0.0627 (3)	1.0796 (3)	0.31969 (11)	0.0203 (6)	
C13B	0.0402 (3)	0.9793 (3)	0.25173 (11)	0.0199 (6)	
C14B	0.1555 (3)	0.9272 (3)	0.15448 (11)	0.0190 (5)	
C15B	0.0435 (3)	0.9761 (3)	0.11971 (11)	0.0205 (6)	
C16B	0.1880 (3)	1.0798 (3)	0.05979 (11)	0.0190 (5)	
C17B	0.3088 (3)	1.0223 (3)	0.09096 (11)	0.0177 (5)	
C18B	0.2900 (3)	0.9505 (3)	0.13989 (11)	0.0179 (5)	
C19B	0.4140 (3)	0.9067 (3)	0.17522 (11)	0.0194 (6)	
C20B	0.5258 (3)	0.7806 (3)	0.17060 (12)	0.0248 (6)	
H20B	0.5217	0.7180	0.1459	0.030*	
C21B	0.6430 (3)	0.7456 (3)	0.20176 (13)	0.0285 (7)	
H21B	0.7179	0.6582	0.1988	0.034*	
C22B	0.6517 (3)	0.8372 (3)	0.23727 (12)	0.0255 (6)	
H22B	0.7320	0.8143	0.2587	0.031*	
C23B	0.5406 (3)	0.9624 (3)	0.24066 (11)	0.0216 (6)	
C24B	0.4199 (3)	0.9991 (3)	0.21079 (11)	0.0214 (6)	
H24B	0.3436	1.0850	0.2147	0.026*	
C25B	−0.1006 (3)	0.9448 (4)	0.13195 (13)	0.0278 (7)	
H25D	−0.1602	0.9789	0.1017	0.042*	
H25E	−0.1519	0.9931	0.1626	0.042*	
H25F	−0.0836	0.8419	0.1395	0.042*	
C26B	0.4452 (3)	1.0496 (3)	0.07378 (11)	0.0209 (6)	
H2NA	0.283 (4)	0.831 (4)	−0.0159 (13)	0.020 (8)*	
H1NA	0.129 (4)	0.852 (4)	−0.0238 (14)	0.026 (9)*	
H2NB	0.116 (4)	1.215 (4)	−0.0027 (16)	0.036 (10)*	
H1NB	0.274 (5)	1.183 (4)	0.0053 (16)	0.040 (11)*	

### Atomic displacement parameters (Å^2^)

**Table:**

	*U*^11^	*U*^22^	*U*^33^	*U*^12^	*U*^13^	*U*^23^
S1A	0.0232 (4)	0.0202 (3)	0.0190 (3)	−0.0060 (3)	−0.0007 (3)	−0.0009 (3)
O1A	0.0226 (11)	0.0401 (13)	0.0190 (10)	−0.0072 (9)	−0.0023 (8)	0.0022 (9)
O2A	0.0244 (10)	0.0202 (10)	0.0169 (10)	−0.0032 (8)	−0.0009 (8)	−0.0007 (8)
O3A	0.0607 (19)	0.080 (2)	0.0346 (15)	−0.0171 (16)	−0.0291 (14)	−0.0020 (14)
O4A	0.0553 (17)	0.0385 (14)	0.0248 (12)	−0.0119 (12)	−0.0011 (11)	−0.0082 (10)
N1A	0.0297 (14)	0.0243 (13)	0.0229 (13)	0.0009 (11)	−0.0020 (11)	−0.0003 (10)
N2A	0.0278 (14)	0.0284 (14)	0.0227 (13)	0.0029 (11)	−0.0025 (11)	−0.0019 (11)
N3A	0.0177 (11)	0.0284 (13)	0.0156 (11)	−0.0074 (10)	−0.0018 (9)	−0.0018 (10)
N4A	0.0155 (12)	0.0284 (13)	0.0199 (12)	−0.0068 (11)	−0.0027 (10)	0.0016 (10)
N5A	0.0200 (13)	0.0388 (15)	0.0268 (14)	−0.0109 (11)	−0.0056 (10)	0.0103 (11)
N6A	0.0445 (17)	0.0364 (16)	0.0245 (14)	−0.0167 (14)	−0.0128 (13)	0.0004 (12)
C1A	0.0199 (14)	0.0292 (16)	0.0268 (15)	−0.0081 (12)	0.0018 (12)	−0.0045 (12)
C2A	0.0186 (14)	0.0261 (15)	0.0338 (17)	−0.0069 (12)	−0.0062 (12)	−0.0014 (13)
C3A	0.0269 (15)	0.0174 (13)	0.0283 (16)	−0.0085 (12)	−0.0057 (12)	−0.0007 (11)
C4A	0.0319 (16)	0.0250 (15)	0.0303 (17)	−0.0124 (13)	−0.0112 (13)	0.0050 (12)
C5A	0.048 (2)	0.0266 (16)	0.0243 (16)	−0.0156 (15)	−0.0109 (14)	0.0040 (13)
C6A	0.0421 (19)	0.0228 (15)	0.0234 (15)	−0.0116 (14)	0.0031 (13)	−0.0011 (12)
C7A	0.0294 (16)	0.0194 (14)	0.0269 (16)	−0.0070 (12)	−0.0010 (12)	−0.0021 (12)
C8A	0.0260 (15)	0.0142 (13)	0.0239 (14)	−0.0078 (11)	−0.0027 (12)	−0.0013 (11)
C9A	0.0213 (14)	0.0180 (13)	0.0248 (15)	−0.0045 (11)	−0.0037 (11)	−0.0006 (11)
C10A	0.0260 (15)	0.0244 (14)	0.0207 (14)	−0.0088 (12)	−0.0034 (12)	−0.0020 (11)
C11A	0.0233 (15)	0.0260 (15)	0.0213 (15)	−0.0039 (12)	−0.0009 (11)	−0.0004 (12)
C12A	0.0185 (14)	0.0236 (14)	0.0211 (14)	−0.0035 (11)	0.0010 (11)	−0.0028 (11)
C13A	0.0175 (13)	0.0229 (14)	0.0165 (13)	−0.0049 (11)	−0.0027 (10)	0.0024 (11)
C14A	0.0181 (13)	0.0233 (14)	0.0182 (13)	−0.0063 (11)	0.0007 (11)	−0.0032 (11)
C15A	0.0183 (13)	0.0281 (15)	0.0158 (13)	−0.0064 (11)	−0.0006 (11)	−0.0009 (11)
C16A	0.0180 (13)	0.0254 (14)	0.0130 (12)	−0.0056 (11)	−0.0019 (10)	−0.0025 (11)
C17A	0.0153 (13)	0.0271 (15)	0.0151 (13)	−0.0054 (11)	−0.0018 (10)	−0.0033 (11)
C18A	0.0176 (13)	0.0210 (13)	0.0147 (13)	−0.0028 (11)	−0.0001 (10)	−0.0051 (10)
C19A	0.0182 (13)	0.0212 (14)	0.0202 (14)	−0.0069 (11)	−0.0023 (11)	−0.0009 (11)
C20A	0.0233 (15)	0.0243 (15)	0.0272 (16)	−0.0036 (12)	0.0009 (12)	−0.0021 (12)
C21A	0.0183 (14)	0.0247 (15)	0.0390 (18)	−0.0007 (12)	−0.0009 (13)	0.0015 (13)
C22A	0.0235 (15)	0.0277 (16)	0.0376 (18)	−0.0058 (13)	−0.0127 (13)	0.0064 (13)
C23A	0.0317 (16)	0.0261 (15)	0.0206 (14)	−0.0120 (13)	−0.0065 (12)	0.0008 (12)
C24A	0.0205 (14)	0.0206 (14)	0.0208 (14)	−0.0070 (11)	−0.0019 (11)	−0.0009 (11)
C25A	0.0219 (15)	0.0364 (18)	0.0287 (16)	−0.0115 (13)	−0.0066 (12)	0.0064 (13)
C26A	0.0206 (14)	0.0263 (15)	0.0147 (13)	−0.0039 (12)	−0.0049 (11)	0.0025 (11)
S1B	0.0244 (4)	0.0207 (3)	0.0181 (3)	−0.0055 (3)	0.0014 (3)	0.0021 (3)
O1B	0.0316 (12)	0.0241 (11)	0.0218 (11)	−0.0076 (9)	0.0063 (9)	−0.0027 (8)
O2B	0.0193 (10)	0.0233 (10)	0.0165 (10)	−0.0022 (8)	−0.0020 (8)	0.0013 (8)
O3B	0.0428 (15)	0.0419 (15)	0.0556 (17)	−0.0091 (12)	−0.0321 (13)	−0.0043 (13)
O4B	0.0333 (13)	0.0393 (14)	0.0402 (14)	−0.0031 (11)	−0.0077 (11)	−0.0170 (11)
N1B	0.0301 (14)	0.0273 (14)	0.0220 (13)	−0.0054 (11)	0.0050 (11)	−0.0027 (10)
N2B	0.0308 (14)	0.0245 (13)	0.0201 (13)	−0.0062 (11)	0.0053 (10)	−0.0003 (10)
N3B	0.0139 (11)	0.0262 (12)	0.0172 (11)	−0.0054 (9)	−0.0023 (9)	−0.0011 (9)
N4B	0.0155 (12)	0.0376 (15)	0.0205 (13)	−0.0103 (11)	−0.0027 (10)	0.0072 (11)
N5B	0.0196 (13)	0.0344 (15)	0.0262 (14)	−0.0087 (11)	−0.0045 (10)	0.0051 (11)
N6B	0.0297 (14)	0.0279 (14)	0.0242 (13)	−0.0101 (11)	−0.0101 (11)	0.0018 (11)
C1B	0.0180 (13)	0.0185 (13)	0.0245 (15)	−0.0046 (11)	0.0003 (11)	−0.0027 (11)
C2B	0.0177 (13)	0.0220 (14)	0.0264 (15)	−0.0077 (11)	−0.0012 (11)	−0.0071 (12)
C3B	0.0231 (14)	0.0227 (14)	0.0234 (15)	−0.0058 (12)	−0.0017 (12)	−0.0050 (12)
C4B	0.0273 (15)	0.0292 (16)	0.0221 (15)	−0.0112 (13)	−0.0034 (12)	−0.0005 (12)
C5B	0.0313 (17)	0.0372 (18)	0.0213 (15)	−0.0121 (14)	0.0022 (12)	−0.0097 (13)
C6B	0.0275 (15)	0.0256 (15)	0.0289 (16)	−0.0095 (12)	0.0036 (12)	−0.0127 (13)
C7B	0.0173 (13)	0.0221 (14)	0.0288 (15)	−0.0061 (11)	0.0019 (11)	−0.0075 (12)
C8B	0.0234 (15)	0.0197 (14)	0.0354 (17)	−0.0063 (12)	0.0015 (13)	−0.0059 (12)
C9B	0.0232 (15)	0.0204 (14)	0.0311 (16)	−0.0069 (12)	0.0016 (12)	0.0016 (12)
C10B	0.0199 (14)	0.0253 (15)	0.0212 (14)	−0.0074 (11)	0.0043 (11)	−0.0027 (11)
C11B	0.0239 (14)	0.0249 (15)	0.0202 (14)	−0.0077 (12)	0.0000 (11)	−0.0004 (11)
C12B	0.0169 (13)	0.0254 (14)	0.0185 (14)	−0.0064 (11)	−0.0008 (10)	−0.0012 (11)
C13B	0.0188 (13)	0.0257 (14)	0.0150 (13)	−0.0086 (11)	0.0003 (10)	0.0029 (11)
C14B	0.0193 (13)	0.0188 (13)	0.0170 (13)	−0.0032 (11)	−0.0003 (11)	−0.0010 (10)
C15B	0.0201 (14)	0.0216 (14)	0.0201 (14)	−0.0070 (11)	0.0003 (11)	−0.0022 (11)
C16B	0.0162 (13)	0.0237 (14)	0.0179 (13)	−0.0065 (11)	−0.0015 (10)	−0.0029 (11)
C17B	0.0132 (12)	0.0199 (13)	0.0188 (13)	−0.0033 (10)	−0.0008 (10)	−0.0016 (10)
C18B	0.0191 (13)	0.0155 (12)	0.0177 (13)	−0.0026 (10)	−0.0024 (10)	−0.0019 (10)
C19B	0.0175 (13)	0.0220 (14)	0.0191 (13)	−0.0084 (11)	−0.0005 (11)	0.0028 (11)
C20B	0.0231 (15)	0.0236 (15)	0.0262 (15)	−0.0051 (12)	−0.0033 (12)	0.0000 (12)
C21B	0.0208 (15)	0.0264 (16)	0.0339 (17)	−0.0004 (12)	−0.0049 (13)	0.0000 (13)
C22B	0.0181 (14)	0.0305 (16)	0.0250 (15)	−0.0050 (12)	−0.0065 (11)	0.0077 (12)
C23B	0.0235 (14)	0.0252 (14)	0.0164 (13)	−0.0077 (12)	−0.0044 (11)	0.0008 (11)
C24B	0.0193 (14)	0.0215 (14)	0.0204 (14)	−0.0021 (11)	−0.0045 (11)	0.0024 (11)
C25B	0.0237 (15)	0.0347 (17)	0.0258 (16)	−0.0122 (13)	−0.0032 (12)	0.0052 (13)
C26B	0.0191 (14)	0.0230 (14)	0.0186 (14)	−0.0036 (11)	−0.0053 (11)	0.0030 (11)

### Geometric parameters (Å, º)

**Table:**

S1A—C13A	1.746 (3)	S1B—C13B	1.742 (3)
S1A—C14A	1.779 (3)	S1B—C14B	1.773 (3)
O1A—C10A	1.378 (4)	O1B—C10B	1.381 (4)
O1A—C11A	1.414 (4)	O1B—C11B	1.409 (4)
O2A—C13A	1.361 (3)	O2B—C12B	1.370 (4)
O2A—C12A	1.366 (3)	O2B—C13B	1.374 (3)
O3A—N6A	1.216 (4)	O3B—N6B	1.224 (3)
O4A—N6A	1.219 (4)	O4B—N6B	1.221 (4)
N1A—C12A	1.285 (4)	N1B—C12B	1.273 (4)
N1A—N2A	1.416 (4)	N1B—N2B	1.426 (4)
N2A—C13A	1.287 (4)	N2B—C13B	1.287 (4)
N3A—C15A	1.341 (4)	N3B—C15B	1.334 (4)
N3A—C16A	1.346 (4)	N3B—C16B	1.359 (3)
N4A—C16A	1.342 (4)	N4B—C16B	1.334 (4)
N4A—H2NA	0.86 (4)	N4B—H2NB	0.92 (4)
N4A—H1NA	0.84 (4)	N4B—H1NB	0.85 (4)
N5A—C26A	1.149 (4)	N5B—C26B	1.149 (4)
N6A—C23A	1.479 (4)	N6B—C23B	1.471 (4)
C1A—C2A	1.361 (4)	C1B—C10B	1.374 (4)
C1A—C10A	1.413 (4)	C1B—C2B	1.421 (4)
C1A—H1AA	0.9500	C1B—H1BA	0.9500
C2A—C3A	1.417 (4)	C2B—C3B	1.410 (4)
C2A—H2AA	0.9500	C2B—C7B	1.424 (4)
C3A—C4A	1.415 (4)	C3B—C4B	1.374 (4)
C3A—C8A	1.422 (4)	C3B—H3BA	0.9500
C4A—C5A	1.375 (5)	C4B—C5B	1.414 (5)
C4A—H4AA	0.9500	C4B—H4BA	0.9500
C5A—C6A	1.398 (5)	C5B—C6B	1.368 (5)
C5A—H5AA	0.9500	C5B—H5BA	0.9500
C6A—C7A	1.378 (4)	C6B—C7B	1.416 (4)
C6A—H6AA	0.9500	C6B—H6BA	0.9500
C7A—C8A	1.416 (4)	C7B—C8B	1.410 (4)
C7A—H7AA	0.9500	C8B—C9B	1.363 (5)
C8A—C9A	1.419 (4)	C8B—H8BA	0.9500
C9A—C10A	1.362 (4)	C9B—C10B	1.420 (4)
C9A—H9AA	0.9500	C9B—H9BA	0.9500
C11A—C12A	1.477 (4)	C11B—C12B	1.489 (4)
C11A—H11A	0.9900	C11B—H11C	0.9900
C11A—H11B	0.9900	C11B—H11D	0.9900
C14A—C18A	1.397 (4)	C14B—C18B	1.388 (4)
C14A—C15A	1.405 (4)	C14B—C15B	1.405 (4)
C15A—C25A	1.499 (4)	C15B—C25B	1.503 (4)
C16A—C17A	1.425 (4)	C16B—C17B	1.415 (4)
C17A—C18A	1.395 (4)	C17B—C18B	1.393 (4)
C17A—C26A	1.434 (4)	C17B—C26B	1.432 (4)
C18A—C19A	1.495 (4)	C18B—C19B	1.501 (4)
C19A—C24A	1.387 (4)	C19B—C24B	1.383 (4)
C19A—C20A	1.395 (4)	C19B—C20B	1.389 (4)
C20A—C21A	1.394 (4)	C20B—C21B	1.385 (4)
C20A—H20A	0.9500	C20B—H20B	0.9500
C21A—C22A	1.391 (5)	C21B—C22B	1.386 (5)
C21A—H21A	0.9500	C21B—H21B	0.9500
C22A—C23A	1.376 (5)	C22B—C23B	1.377 (4)
C22A—H22A	0.9500	C22B—H22B	0.9500
C23A—C24A	1.385 (4)	C23B—C24B	1.391 (4)
C24A—H24A	0.9500	C24B—H24B	0.9500
C25A—H25A	0.9800	C25B—H25D	0.9800
C25A—H25B	0.9800	C25B—H25E	0.9800
C25A—H25C	0.9800	C25B—H25F	0.9800
			
C13A—S1A—C14A	99.92 (13)	C13B—S1B—C14B	98.96 (13)
C10A—O1A—C11A	116.0 (2)	C10B—O1B—C11B	115.3 (2)
C13A—O2A—C12A	101.6 (2)	C12B—O2B—C13B	101.0 (2)
C12A—N1A—N2A	105.9 (2)	C12B—N1B—N2B	106.1 (2)
C13A—N2A—N1A	105.5 (2)	C13B—N2B—N1B	105.2 (2)
C15A—N3A—C16A	119.5 (2)	C15B—N3B—C16B	119.2 (2)
C16A—N4A—H2NA	119 (2)	C16B—N4B—H2NB	125 (2)
C16A—N4A—H1NA	119 (2)	C16B—N4B—H1NB	119 (3)
H2NA—N4A—H1NA	119 (3)	H2NB—N4B—H1NB	115 (4)
O3A—N6A—O4A	124.7 (3)	O4B—N6B—O3B	124.0 (3)
O3A—N6A—C23A	117.7 (3)	O4B—N6B—C23B	118.3 (2)
O4A—N6A—C23A	117.6 (3)	O3B—N6B—C23B	117.7 (3)
C2A—C1A—C10A	119.6 (3)	C10B—C1B—C2B	119.6 (3)
C2A—C1A—H1AA	120.2	C10B—C1B—H1BA	120.2
C10A—C1A—H1AA	120.2	C2B—C1B—H1BA	120.2
C1A—C2A—C3A	121.5 (3)	C3B—C2B—C1B	121.4 (3)
C1A—C2A—H2AA	119.2	C3B—C2B—C7B	118.9 (3)
C3A—C2A—H2AA	119.2	C1B—C2B—C7B	119.7 (3)
C4A—C3A—C2A	122.3 (3)	C4B—C3B—C2B	121.3 (3)
C4A—C3A—C8A	119.5 (3)	C4B—C3B—H3BA	119.4
C2A—C3A—C8A	118.2 (3)	C2B—C3B—H3BA	119.4
C5A—C4A—C3A	120.1 (3)	C3B—C4B—C5B	119.7 (3)
C5A—C4A—H4AA	120.0	C3B—C4B—H4BA	120.1
C3A—C4A—H4AA	120.0	C5B—C4B—H4BA	120.1
C4A—C5A—C6A	120.7 (3)	C6B—C5B—C4B	120.2 (3)
C4A—C5A—H5AA	119.7	C6B—C5B—H5BA	119.9
C6A—C5A—H5AA	119.7	C4B—C5B—H5BA	119.9
C7A—C6A—C5A	120.5 (3)	C5B—C6B—C7B	121.2 (3)
C7A—C6A—H6AA	119.8	C5B—C6B—H6BA	119.4
C5A—C6A—H6AA	119.8	C7B—C6B—H6BA	119.4
C6A—C7A—C8A	120.5 (3)	C8B—C7B—C6B	123.1 (3)
C6A—C7A—H7AA	119.8	C8B—C7B—C2B	118.3 (3)
C8A—C7A—H7AA	119.8	C6B—C7B—C2B	118.6 (3)
C7A—C8A—C9A	121.7 (3)	C9B—C8B—C7B	121.9 (3)
C7A—C8A—C3A	118.7 (3)	C9B—C8B—H8BA	119.1
C9A—C8A—C3A	119.6 (3)	C7B—C8B—H8BA	119.1
C10A—C9A—C8A	119.8 (3)	C8B—C9B—C10B	119.5 (3)
C10A—C9A—H9AA	120.1	C8B—C9B—H9BA	120.3
C8A—C9A—H9AA	120.1	C10B—C9B—H9BA	120.3
C9A—C10A—O1A	124.5 (3)	C1B—C10B—O1B	124.6 (3)
C9A—C10A—C1A	121.2 (3)	C1B—C10B—C9B	121.0 (3)
O1A—C10A—C1A	114.3 (3)	O1B—C10B—C9B	114.4 (3)
O1A—C11A—C12A	108.0 (2)	O1B—C11B—C12B	108.9 (2)
O1A—C11A—H11A	110.1	O1B—C11B—H11C	109.9
C12A—C11A—H11A	110.1	C12B—C11B—H11C	109.9
O1A—C11A—H11B	110.1	O1B—C11B—H11D	109.9
C12A—C11A—H11B	110.1	C12B—C11B—H11D	109.9
H11A—C11A—H11B	108.4	H11C—C11B—H11D	108.3
N1A—C12A—O2A	113.3 (3)	N1B—C12B—O2B	113.9 (2)
N1A—C12A—C11A	127.7 (3)	N1B—C12B—C11B	132.0 (3)
O2A—C12A—C11A	118.9 (2)	O2B—C12B—C11B	114.1 (2)
N2A—C13A—O2A	113.7 (3)	N2B—C13B—O2B	113.7 (3)
N2A—C13A—S1A	129.3 (2)	N2B—C13B—S1B	130.8 (2)
O2A—C13A—S1A	117.1 (2)	O2B—C13B—S1B	115.5 (2)
C18A—C14A—C15A	119.7 (3)	C18B—C14B—C15B	119.3 (3)
C18A—C14A—S1A	120.4 (2)	C18B—C14B—S1B	120.1 (2)
C15A—C14A—S1A	119.8 (2)	C15B—C14B—S1B	120.6 (2)
N3A—C15A—C14A	122.1 (3)	N3B—C15B—C14B	122.4 (3)
N3A—C15A—C25A	115.9 (2)	N3B—C15B—C25B	116.2 (2)
C14A—C15A—C25A	122.0 (3)	C14B—C15B—C25B	121.4 (3)
N4A—C16A—N3A	116.6 (2)	N4B—C16B—N3B	116.8 (2)
N4A—C16A—C17A	122.3 (3)	N4B—C16B—C17B	122.3 (3)
N3A—C16A—C17A	121.1 (3)	N3B—C16B—C17B	120.8 (3)
C18A—C17A—C16A	119.6 (3)	C18B—C17B—C16B	119.5 (2)
C18A—C17A—C26A	121.6 (2)	C18B—C17B—C26B	120.8 (2)
C16A—C17A—C26A	118.7 (3)	C16B—C17B—C26B	119.4 (2)
C17A—C18A—C14A	117.8 (3)	C14B—C18B—C17B	118.3 (2)
C17A—C18A—C19A	119.9 (2)	C14B—C18B—C19B	123.0 (2)
C14A—C18A—C19A	122.1 (3)	C17B—C18B—C19B	118.7 (2)
C24A—C19A—C20A	119.4 (3)	C24B—C19B—C20B	120.0 (3)
C24A—C19A—C18A	118.8 (2)	C24B—C19B—C18B	118.5 (2)
C20A—C19A—C18A	121.7 (3)	C20B—C19B—C18B	121.4 (3)
C19A—C20A—C21A	120.1 (3)	C21B—C20B—C19B	120.5 (3)
C19A—C20A—H20A	119.9	C21B—C20B—H20B	119.7
C21A—C20A—H20A	119.9	C19B—C20B—H20B	119.7
C22A—C21A—C20A	120.8 (3)	C22B—C21B—C20B	120.4 (3)
C22A—C21A—H21A	119.6	C22B—C21B—H21B	119.8
C20A—C21A—H21A	119.6	C20B—C21B—H21B	119.8
C23A—C22A—C21A	117.7 (3)	C23B—C22B—C21B	118.1 (3)
C23A—C22A—H22A	121.1	C23B—C22B—H22B	121.0
C21A—C22A—H22A	121.1	C21B—C22B—H22B	121.0
C22A—C23A—C24A	122.9 (3)	C22B—C23B—C24B	122.9 (3)
C22A—C23A—N6A	119.6 (3)	C22B—C23B—N6B	119.3 (3)
C24A—C23A—N6A	117.4 (3)	C24B—C23B—N6B	117.8 (3)
C23A—C24A—C19A	119.1 (3)	C19B—C24B—C23B	118.1 (3)
C23A—C24A—H24A	120.5	C19B—C24B—H24B	120.9
C19A—C24A—H24A	120.5	C23B—C24B—H24B	120.9
C15A—C25A—H25A	109.5	C15B—C25B—H25D	109.5
C15A—C25A—H25B	109.5	C15B—C25B—H25E	109.5
H25A—C25A—H25B	109.5	H25D—C25B—H25E	109.5
C15A—C25A—H25C	109.5	C15B—C25B—H25F	109.5
H25A—C25A—H25C	109.5	H25D—C25B—H25F	109.5
H25B—C25A—H25C	109.5	H25E—C25B—H25F	109.5
N5A—C26A—C17A	177.2 (3)	N5B—C26B—C17B	178.0 (3)
			
C12A—N1A—N2A—C13A	−0.5 (3)	C12B—N1B—N2B—C13B	0.4 (3)
C10A—C1A—C2A—C3A	−1.2 (5)	C10B—C1B—C2B—C3B	178.3 (3)
C1A—C2A—C3A—C4A	−176.6 (3)	C10B—C1B—C2B—C7B	−0.8 (4)
C1A—C2A—C3A—C8A	1.1 (4)	C1B—C2B—C3B—C4B	−179.9 (3)
C2A—C3A—C4A—C5A	178.8 (3)	C7B—C2B—C3B—C4B	−0.8 (4)
C8A—C3A—C4A—C5A	1.1 (4)	C2B—C3B—C4B—C5B	−0.7 (5)
C3A—C4A—C5A—C6A	1.7 (5)	C3B—C4B—C5B—C6B	1.1 (5)
C4A—C5A—C6A—C7A	−2.5 (5)	C4B—C5B—C6B—C7B	−0.1 (5)
C5A—C6A—C7A—C8A	0.5 (5)	C5B—C6B—C7B—C8B	178.8 (3)
C6A—C7A—C8A—C9A	−178.0 (3)	C5B—C6B—C7B—C2B	−1.4 (4)
C6A—C7A—C8A—C3A	2.3 (4)	C3B—C2B—C7B—C8B	−178.4 (3)
C4A—C3A—C8A—C7A	−3.1 (4)	C1B—C2B—C7B—C8B	0.7 (4)
C2A—C3A—C8A—C7A	179.1 (3)	C3B—C2B—C7B—C6B	1.9 (4)
C4A—C3A—C8A—C9A	177.2 (3)	C1B—C2B—C7B—C6B	−179.0 (3)
C2A—C3A—C8A—C9A	−0.6 (4)	C6B—C7B—C8B—C9B	179.1 (3)
C7A—C8A—C9A—C10A	−179.5 (3)	C2B—C7B—C8B—C9B	−0.6 (4)
C3A—C8A—C9A—C10A	0.1 (4)	C7B—C8B—C9B—C10B	0.5 (5)
C8A—C9A—C10A—O1A	179.7 (3)	C2B—C1B—C10B—O1B	179.9 (3)
C8A—C9A—C10A—C1A	−0.2 (4)	C2B—C1B—C10B—C9B	0.7 (4)
C11A—O1A—C10A—C9A	−3.9 (4)	C11B—O1B—C10B—C1B	12.9 (4)
C11A—O1A—C10A—C1A	176.0 (3)	C11B—O1B—C10B—C9B	−167.9 (3)
C2A—C1A—C10A—C9A	0.8 (5)	C8B—C9B—C10B—C1B	−0.6 (4)
C2A—C1A—C10A—O1A	−179.2 (3)	C8B—C9B—C10B—O1B	−179.8 (3)
C10A—O1A—C11A—C12A	−179.1 (2)	C10B—O1B—C11B—C12B	−176.4 (2)
N2A—N1A—C12A—O2A	1.3 (3)	N2B—N1B—C12B—O2B	−0.3 (3)
N2A—N1A—C12A—C11A	−174.9 (3)	N2B—N1B—C12B—C11B	179.0 (3)
C13A—O2A—C12A—N1A	−1.5 (3)	C13B—O2B—C12B—N1B	0.2 (3)
C13A—O2A—C12A—C11A	175.1 (3)	C13B—O2B—C12B—C11B	−179.3 (2)
O1A—C11A—C12A—N1A	−136.7 (3)	O1B—C11B—C12B—N1B	−8.7 (4)
O1A—C11A—C12A—O2A	47.3 (4)	O1B—C11B—C12B—O2B	170.6 (2)
N1A—N2A—C13A—O2A	−0.4 (3)	N1B—N2B—C13B—O2B	−0.3 (3)
N1A—N2A—C13A—S1A	−178.7 (2)	N1B—N2B—C13B—S1B	−179.0 (2)
C12A—O2A—C13A—N2A	1.1 (3)	C12B—O2B—C13B—N2B	0.1 (3)
C12A—O2A—C13A—S1A	179.6 (2)	C12B—O2B—C13B—S1B	179.02 (19)
C14A—S1A—C13A—N2A	36.7 (3)	C14B—S1B—C13B—N2B	−2.7 (3)
C14A—S1A—C13A—O2A	−141.5 (2)	C14B—S1B—C13B—O2B	178.5 (2)
C13A—S1A—C14A—C18A	94.8 (2)	C13B—S1B—C14B—C18B	98.0 (2)
C13A—S1A—C14A—C15A	−89.0 (2)	C13B—S1B—C14B—C15B	−83.0 (2)
C16A—N3A—C15A—C14A	3.2 (4)	C16B—N3B—C15B—C14B	2.7 (4)
C16A—N3A—C15A—C25A	−177.4 (3)	C16B—N3B—C15B—C25B	−177.3 (3)
C18A—C14A—C15A—N3A	−0.6 (4)	C18B—C14B—C15B—N3B	−4.4 (4)
S1A—C14A—C15A—N3A	−176.8 (2)	S1B—C14B—C15B—N3B	176.5 (2)
C18A—C14A—C15A—C25A	180.0 (3)	C18B—C14B—C15B—C25B	175.7 (3)
S1A—C14A—C15A—C25A	3.8 (4)	S1B—C14B—C15B—C25B	−3.4 (4)
C15A—N3A—C16A—N4A	178.0 (3)	C15B—N3B—C16B—N4B	−177.0 (3)
C15A—N3A—C16A—C17A	−2.3 (4)	C15B—N3B—C16B—C17B	3.1 (4)
N4A—C16A—C17A—C18A	178.5 (3)	N4B—C16B—C17B—C18B	172.8 (3)
N3A—C16A—C17A—C18A	−1.2 (4)	N3B—C16B—C17B—C18B	−7.3 (4)
N4A—C16A—C17A—C26A	−0.6 (4)	N4B—C16B—C17B—C26B	−1.8 (4)
N3A—C16A—C17A—C26A	179.7 (3)	N3B—C16B—C17B—C26B	178.2 (3)
C16A—C17A—C18A—C14A	3.7 (4)	C15B—C14B—C18B—C17B	0.1 (4)
C26A—C17A—C18A—C14A	−177.2 (3)	S1B—C14B—C18B—C17B	179.2 (2)
C16A—C17A—C18A—C19A	−171.9 (2)	C15B—C14B—C18B—C19B	177.7 (3)
C26A—C17A—C18A—C19A	7.2 (4)	S1B—C14B—C18B—C19B	−3.2 (4)
C15A—C14A—C18A—C17A	−2.9 (4)	C16B—C17B—C18B—C14B	5.5 (4)
S1A—C14A—C18A—C17A	173.3 (2)	C26B—C17B—C18B—C14B	180.0 (3)
C15A—C14A—C18A—C19A	172.7 (3)	C16B—C17B—C18B—C19B	−172.3 (3)
S1A—C14A—C18A—C19A	−11.2 (4)	C26B—C17B—C18B—C19B	2.2 (4)
C17A—C18A—C19A—C24A	111.4 (3)	C14B—C18B—C19B—C24B	−86.1 (3)
C14A—C18A—C19A—C24A	−64.0 (4)	C17B—C18B—C19B—C24B	91.5 (3)
C17A—C18A—C19A—C20A	−64.7 (4)	C14B—C18B—C19B—C20B	97.7 (3)
C14A—C18A—C19A—C20A	119.8 (3)	C17B—C18B—C19B—C20B	−84.6 (3)
C24A—C19A—C20A—C21A	0.8 (4)	C24B—C19B—C20B—C21B	0.6 (4)
C18A—C19A—C20A—C21A	176.9 (3)	C18B—C19B—C20B—C21B	176.7 (3)
C19A—C20A—C21A—C22A	−0.9 (5)	C19B—C20B—C21B—C22B	−1.2 (5)
C20A—C21A—C22A—C23A	0.8 (5)	C20B—C21B—C22B—C23B	0.4 (5)
C21A—C22A—C23A—C24A	−0.5 (5)	C21B—C22B—C23B—C24B	1.2 (5)
C21A—C22A—C23A—N6A	−177.4 (3)	C21B—C22B—C23B—N6B	−177.0 (3)
O3A—N6A—C23A—C22A	−1.7 (5)	O4B—N6B—C23B—C22B	−173.0 (3)
O4A—N6A—C23A—C22A	177.7 (3)	O3B—N6B—C23B—C22B	8.5 (4)
O3A—N6A—C23A—C24A	−178.8 (3)	O4B—N6B—C23B—C24B	8.7 (4)
O4A—N6A—C23A—C24A	0.6 (4)	O3B—N6B—C23B—C24B	−169.7 (3)
C22A—C23A—C24A—C19A	0.4 (5)	C20B—C19B—C24B—C23B	0.8 (4)
N6A—C23A—C24A—C19A	177.3 (3)	C18B—C19B—C24B—C23B	−175.3 (3)
C20A—C19A—C24A—C23A	−0.5 (4)	C22B—C23B—C24B—C19B	−1.8 (4)
C18A—C19A—C24A—C23A	−176.7 (3)	N6B—C23B—C24B—C19B	176.4 (3)

### Hydrogen-bond geometry (Å, º)

Cg1, Cg2, Cg3 and Cg4 are the centroids of the C1B/C2B/C7B–C10B, C2B–C7B, C1A/C2A/C3A/C8A–C10A and O2A/C12A/N1A/N2A/C13A rings, respecively.

**Table:**

*D*—H···*A*	*D*—H	H···*A*	*D*···*A*	*D*—H···*A*
N4*A*—H2*NA*···N5*B*^i^	0.86 (4)	2.21 (4)	3.025 (4)	158 (3)
N4*A*—H1*NA*···N3*B*^ii^	0.84 (4)	2.23 (4)	3.056 (4)	165 (4)
N4*B*—H2*NB*···N3*A*^ii^	0.92 (4)	2.08 (4)	2.991 (4)	174 (4)
N4*B*—H1*NB*···N5*A*^i^	0.85 (5)	2.34 (5)	3.157 (4)	162 (4)
C11*A*—H11*B*···O1*B*^iii^	0.99	2.36	3.332 (4)	168
C11*B*—H11*C*···O3*B*^iv^	0.99	2.60	3.173 (4)	117
C21*B*—H21*B*···N2*A*^v^	0.95	2.54	3.333 (4)	142
C22*A*—H22*A*···N1*B*^vi^	0.95	2.60	3.540 (4)	169
C2*A*—H2*AA*···*Cg*1^vi^	0.95	2.78	3.492 (3)	133
C4*A*—H4*AA*···*Cg*2^vi^	0.95	2.62	3.457 (4)	147
C7*A*—H7*AA*···*Cg*1^iii^	0.95	2.80	3.557 (3)	138
C3*B*—H3*BA*···*Cg*3^vii^	0.95	2.67	3.440 (3)	138
C8*B*—H8*BA*···*Cg*3^viii^	0.95	2.83	3.599 (3)	139
C24*A*—H24*A*···*Cg*4	0.95	2.92	3.652 (3)	135

Symmetry codes: (i) −*x*+1, −*y*+2, −*z*; (ii) −*x*, −*y*+2, −*z*; (iii) *x*, *y*−1, *z*; (iv) *x*−1, *y*, *z*; (v) *x*+1, *y*, *z*; (vi) *x*+1, *y*−1, *z*; (vii) −*x*, −*y*+1, −*z*+1; (viii) −*x*, −*y*+2, −*z*+1.
